# High Prevalence of Microsporidia in the North African Hedgehog (*Atelerix algirus*) in the Canary Islands, Spain

**DOI:** 10.3390/ani13111756

**Published:** 2023-05-25

**Authors:** Edgar Baz-González, Néstor Abreu-Acosta, Pilar Foronda

**Affiliations:** 1 Department Obstetricia y Ginecología, Pediatría, Medicina Preventiva y Salud Pública, Toxicología, Medicina Legal y Forense y Parasitología, Universidad de La Laguna (ULL), Avda. Astrofísico F. Sánchez s/n, 38203 San Cristóbal de La Laguna, Tenerife, Canary Islands, Spain; ebazgonz@ull.edu.es; 2 Instituto Universitario de Enfermedades Tropicales y Salud Pública de Canarias (IUETSPC), Universidad de La Laguna (ULL), Avda. Astrofísico F. Sánchez s/n, 38203 San Cristóbal de La Laguna, Tenerife, Canary Islands, Spain; gerencia@nertalab.es; 3 Programa de Doctorado en Ciencias Médicas y Farmacéuticas, Desarrollo y Calidad de Vida, Universidad de La Laguna (ULL), Avda. Astrofísico F. Sánchez s/n, 38203 San Cristóbal de La Laguna, Tenerife, Canary Islands, Spain; 4 Nertalab S.L.U., 38001 Santa Cruz de Tenerife, Tenerife, Canary Islands, Spain

**Keywords:** microsporidia, *Enterocytozoon bieneusi*, *Encephalitozoon cuniculi*, hedgehog, *Atelerix algirus*, zoonotic, Canary Islands

## Abstract

**Simple Summary:**

It is well known that mammals can harbor various pathogens that can affect humans (known as zoonotic pathogens) including viruses, bacteria, fungi and parasites. Microsporidia are a group of pathogens related to fungi and parasites of several animals that can cause diarrhea or systemic infection in humans. Due to the limited knowledge about microsporidia infection in hedgehogs worldwide, this study aimed to analyze the presence and identity of microsporidia in a group of North African hedgehogs from the Canary Islands (Spain). *Enterocytozoon bieneusi* and *Encephalitozoon cuniculi*, two zoonotic species of microsporidia, were identified. These results suggest that microsporidia species with zoonotic risk circulate in the archipelago.

**Abstract:**

Microsporidia are unicellular eukaryotic obligate intracellular parasites with a wide range of hosts reported worldwide; however, little is known about the epidemiological data on microsporidia infection in animals from the Canary Islands. Since data on microsporidia infection in hedgehog species are scarce, the aim of this study was to analyze the presence and identity of microsporidia in a group of North African hedgehogs (*Atelerix algirus*) using microscopic and molecular methods. From December 2020 to September 2021, a total of 36 fecal samples were collected from naturally deceased hedgehogs from Tenerife and Gran Canaria. All samples showed spore-compatible structures (100%; 36/36) under microscopic analysis, of which 61.1% (22/36) were amplified via the nested-polymerase chain reaction (PCR) targeting the partial sequence of the 16S rRNA gene, the internal transcribed spacer (ITS) region, and the partial sequence of the 5.8S rRNA gene. After Sanger sequencing and ITS analysis, *Enterocytozoon bieneusi* was detected in 47.2% (17/36) of the samples, identifying two novel genotypes (AAE1 and AAE2), followed by the detection of an undetermined species in 8.3% (3/36) and *Encephalitozoon cuniculi* genotype I in 5.6% (2/36) of the samples. This study constitutes the first report of microsporidia species in *Atelerix algirus* worldwide, highlighting the high prevalence of zoonotic species.

## 1. Introduction

Microsporidia are unicellular eukaryotic obligate intracellular parasites, spore-forming, and phylogenetically related to the fungi kingdom. Seventeen species have been described as human-pathogenic microsporidia, especially in immunocompromised individuals, of which *Enterocytozoon bieneusi* and the genus *Encephalitozoon* are the most frequent [1].

*Enterocytozoon bieneusi*, with approximately 500 genotypes described based on the sequence analysis of the internal transcribed spacer (ITS) of the rRNA gene, is clustered within 11 phylogenetic groups. Group 1 and Group 2 are considered zoonotic, while the remaining groups (3–11) are considered host-specific [2]. In *Encephalitozoon cuniculi*, four genotypes (I–IV) based on the number of 5′-GTTT-3′ repeats in the ITS have been described, all of which have confirmed zoonotic potential [3]. The number of genotypes of *Encephalitozoon hellem* depends on the target for genotyping (ITS, the polar tube protein locus, or other intergenic spacers), suggesting high intraspecies variability [4]. However, there are no genetic differences within the ITS for genotyping the *Encephalitozoon intestinalis* isolates [5].

Spore transmission can occur through ingestion of contaminated water and food, inhalation of contaminated aerosols, contact with infected animals (zoonotic transmission) or persons (anthroponotic transmission). Zoonotic transmission has been supported by the identification of the same genotypes in humans and animals [6,7].

Microsporidia have been found in several hosts, including livestock, companion and wildlife animals worldwide, but little is known about microsporidia infection in hedgehogs [8]. To our knowledge, the detection of *E. bieneusi* has only been reported in an undetermined species of hedgehog [9], in the Amur hedgehog (*Erinaceus amurensis*) [10] and more recently in the African pygmy hedgehog (*Atelerix albiventris*) [11].

In the Canary Islands (13°23′–18°8′ W and 27°37′–29°24′ N), the only hedgehog species recorded is the North African hedgehog (*Atelerix algirus*), an introduced species from Northwest Africa. The distribution of this mammal in Spain includes the Iberian Peninsula, the Balearic and Canary Islands, as well as Ceuta and Melilla. The introduction of this species has been suggested as an anthroponotic introduction from Morocco to Fuerteventura in 1892, and is currently present in Fuerteventura, Lanzarote, Gran Canaria, and Tenerife. Nonetheless, isolated specimens are also known from La Gomera, El Hierro, and La Palma [12,13].

Epidemiological data on microsporidia infection in the fauna of this archipelago are scarce, and there are no data on *A. algirus* as a host for these parasites. Therefore, the present study aimed to investigate the prevalence and identification of microsporidia in fecal samples from hedgehogs on the Canary Islands.

## 2. Materials and Methods

### 2.1. Ethical Agreement

This study was carried out under the agreement of “Consejería de Transición Ecológica, Lucha contra el Cambio Climático y Planificación Territorial” (Gobierno de Canarias) named “Estudio de patógenos en aves migratorias y en especies exóticas en un escenario de cambio climático”, available online in the Official Bulletin of the Canaries (BOC nº 248, 4 December 2020) [14].

### 2.2. Study Area, Sample Collection, and Preparation

**Figure 1:**
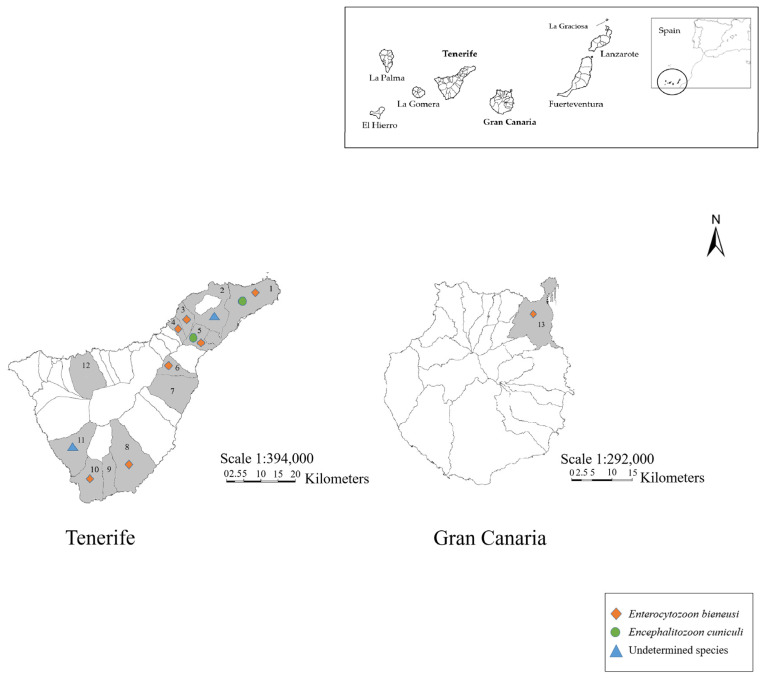
Map of the sampled locations in Tenerife and Gran Canaria. The municipalities are shown in gray—1: Santa Cruz de Tenerife; 2: San Cristóbal de La Laguna; 3: Tacoronte; 4: El Sauzal; 5: El Rosario; 6: Arafo; 7: Güímar; 8: Granadilla de Abona; 9: San Miguel de Abona; 10: Arona; 11: Adeje; 12: Icod de Los Vinos; 13: Las Palmas de Gran Canaria. The symbols indicate samples confirmed via nested-PCR. The original images were taken from Wikimedia Common (https://commons.wikimedia.org/w/index.php?title=File:Mapa_Canarias_municipios.svg&oldid=478721455, accessed on 2 March 2023; https://commons.wikimedia.org/wiki/File:Islas_Canarias_(real_location)_in_Spain.svg, accessed on 2 March 2023) and Gobierno de Canarias (https://www3.gobiernodecanarias.org/medusa/mediateca/ecoescuela/?attachment_id=3333, accessed on 2 March 2023; https://www3.gobiernodecanarias.org/medusa/mediateca/ecoescuela/?attachment_id=3265, accessed on 2 March 2023), in which permission to copy, distribute, or adapt was established. Users: Júlio Reis (https://commons.wikimedia.org/wiki/User:Tintazul, accessed on 2 March 2023), TUBS (https://commons.wikimedia.org/wiki/User:TUBS, accessed on 2 March 2023), GRAFCAN (https://www.grafcan.es/, accessed on 2 March 2023), and IDE Canarias (http://www.idecanarias.es/, accessed on 2 March 2023) (Source: Gobierno de Canarias).

From December 2020 to September 2021, a total of 36 fecal samples were collected by dissecting naturally deceased hedgehogs (*n* = 33) donated by “La Tahonilla” Wildlife Recovery Center in Tenerife, and found dead individuals (*n* = 3) collected by technical personnel of “RedEXOS” in Gran Canaria (Figure 1). For each sampled animal, sex and location were recorded whenever possible (Supplementary Material—Table S1).

All fecal samples were placed in tubes containing 2.5% (*w*/*v*) aqueous potassium dichromate solution (K_2_Cr_2_O_7_) (Merck, Darmstadt, Germany) and stored at 4 °C until further processing.

### 2.3. Staining Method

Fecal samples were stained with Weber’s chromotrope stain (chromotrope 2R [Sigma-Aldrich, St. Louis, MO, USA], and Fast Green [Sigma-Aldrich, St. Louis, MO, USA] and phosphotungstic acid [Sigma-Aldrich, St. Louis, MO, USA]) [15] and microscopically screened for microsporidia spores at a magnification of 1000× under a Leica DM750 microscope model ICC50 HD (Leica Microsystems, Heerbrugg, Switzerland). Samples with spore-compatible structures, ovoid and refractile structures stained pink-red, were considered positive.

### 2.4. DNA Extraction

The total DNA of each fecal sample was extracted using ~500 μL of the sample, previously washed with sterile Phosphate-Buffered Saline (PBS) 1X at room temperature, and centrifuged at 3500 rpm for 15 min to remove the potassium dichromate solution. A commercial FastDNA ^®^ Spin Kit for Soil (MP Biomedicals, Solon, OH, USA) was used following the manufacturer’s instructions, and the homogenizer FastPrep-24^TM^ 5G (MP Biomedicals, Solon, OH, USA) was used as the spore disruptor.

### 2.5. Nested-PCR Amplification

Nested-PCR was carried out in an XP Cycler (Bioer Technology, Hangzhou, China) targeting the partial sequence of the 16S rRNA gene, the complete internal transcribed spacer region (ITS), and the partial sequence of the 5.8S rRNA gene [16].

The amplification reaction of both steps (25 μL) included 0.15 μL of Taq DNA polymerase (5 UI/ μL) (VWR International, Haasrode, Belgium), 2.5 μL of dNTPs mix (200 μM) (Bioline, London, UK), 2.5 μL of 10× key buffer (15 mM Mg^2+^) (VWR International, Haasrode, Belgium), 1.25 μL of MgCl_2_ (25 mM) (VWR International, Haasrode, Belgium), 0.1 μL of each primer and 1 μL of DNA template (or 1 μL of primary PCR product). The primers used were MSP-1, MSP-2A, and MSP2B for the first step and MSP-3, MSP-4A, and MSP4B for the second step. The pairs of primers were used to identify *E. bieneusi* (MSP1/MSP2B—MSP3/MSP4B) and *Encephalitozoon* spp. (MSP1/MSP2A—MSP3/MSP4A) with the following conditions in each reaction: initial denaturation at 94 °C for 3 min, 35 cycles of denaturation at 94 °C for 45 s, annealing at 54 °C for 45 s, and extension at 72 °C for 1 min, and a final step at 72 °C for 7 min [17].

Ten microliters of each PCR product were examined via electrophoresis on 1.5% (*w*/*v*) agarose gels (Fisher Bioreagents, Madrid, Spain) stained with REALSAFE Nucleic Acid Staining Solution (20,000×, REAL, Durviz S.L., Valencia, Spain). An amplified DNA product with sizes between 300 and 500 bp (expected size for *Encephalitozoon* spp. and *E. bieneusi*, respectively) was considered positive and was sequenced using secondary primers.

### 2.6. Sequencing and Phylogenetic Analysis

All nested-PCR positive products were purified using ExoCleanUp FAST (VWR International, Haasrode, Belgium) and sequenced using the Sanger method at the University of La Laguna Sequencing Services (Servicio de Genómica—Servicios Generales de Apoyo a la Investigación de la Universidad de La Laguna, Universidad de La Laguna, Spain).

The obtained sequence chromatograms were analyzed and aligned using the ClustalW program included in MEGA X v10.2.6 (Molecular Evolutionary Genetic Analysis) software (Hachioji, Japan) [18] and compared using the Basic Local Alignment Search Tool (BLAST) in the GenBank database.

Phylogenetic trees were generated using the neighbor-joining method, and genetic distances were calculated using the Kimura 2-parameter model [19,20] with 1000 bootstrap replicates.

Nucleotide sequences were deposited in GenBank under the following accession numbers for *E. bieneusi* (OQ646695–OQ646706; and OQ646730–OQ646734), *E. cuniculi* (OQ646736 and OQ646737) and the undetermined species (OQ646735).

## 3. Results

### 3.1. Light Microscopy

Of the 36 hedgehogs, spore-compatible structures were found in 100% (36/36) of the samples stained with Weber’s chromotrope stain.

### 3.2. Molecular Characterization

A total of 22 (61.1%) samples yielded fragments of the expected sizes (300–500 bp). Sanger sequencing revealed the presence of *E. bieneusi* in 47.2% (17/36) and *E. cuniculi* in 5.6% (2/36) of samples. In addition, three samples (8.3%; 3/36) were identified as undetermined species based on BLAST analysis because of the low homology observed (less than 95%) or because they were not long enough for homology comparison with the reference sequences in MEGA X.

#### 3.2.1. Molecular Characterization of *Enterocytozoon bieneusi*

Genotyping was successful in 94.1% (16/17) of *E. bieneusi*-positive samples. One sequence (5.9%) was not sufficiently long to be genotyped. Two novel genotypes were identified, named AAE1 (*n* = 13) and AAE2 (*n* = 3).

The sequence of the ITS region of genotype AAE1 (242-bp) showed 99.18% homology with genotypes isolated from several mammal species in China: HND-I in snub-nosed monkeys (*Rhinopithecus bieti*) (MK965088.1) and sika deer (*Cervus nippon*) (KX383628.1); and Type IV in raccoon dog (*Nyctereutes procyonoides*) (MN747469.1) and Père David’s deer (*Elaphurus davidianus*) (KP057598.1).

The sequence of the ITS region of the genotype AAE2 (243-bp) showed 99.69% homology with various isolates of WildBoar3 (syn. NCF2, NCF3, NCF4) in the silver fox (*Vulpes vulpes*) and arctic fox (*Vulpes lagopus*) from China (MN029056.1), beech marten (*Martes foina*) (MN218601.1) from Poland, European badger (*Meles meles*) (MG458713.1) from Spain, and red fox (*Vulpes vulpes*) from Poland and Spain (MK256483.1 and MG458714.1, respectively).

The ITS region of genotype AAE1 was 242 bp in length, as a result of the deletion of one nucleotide (position 53), differed by one single nucleotide polymorphism (SNP) compared with genotype HND-I, two SNPs compared with genotype EA1, one SNP and two nucleotide insertions compared with genotype EA2, two SNPs compared with genotype EA3, one SNP compared with EA4, and two SNPs compared with genotype S9. The positions of the SNPs and the insertions are listed in Table 1.

The ITS region of genotype AAE2, 243 bp in length, showed only one SNP at position 104 (G→ A) compared to genotype WildBoar3.

**Figure 2:**
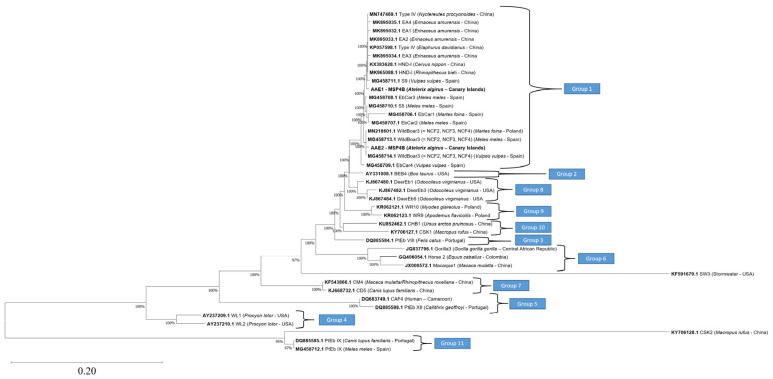
Phylogenetic relationships between the sequence of the internal transcribed spacer region of the rRNA gene of *Enterocytozoon bieneusi* obtained in this study and the sequences of known genotypes deposited in GenBank. The tree was constructed using the neighbor-joining method based on the genetic distance calculated using the Kimura 2-parameter model. Representative sequences from each phylogenetic Group of *E. bieneusi* genotypes (Groups 1–11) were used. Accession numbers are shown in bold, and information regarding the host species and origin are shown in parentheses. There were a total of 239 positions in the final dataset.

The novel genotypes clustered within Group 1 based on phylogenetic analysis (Figure 2). Genotype AAE1 fell into an independent clade close to two clades, one of which was formed by the genotypes isolated from the Amur hedgehog (*E. amurensis*) in China (EA1–EA4) (bootstrap value of 100%). Genotype AAE2 clustered in a clade with the genotype WildBoar3 (syn. NCF2, NCF3, NCF4), which have been previously detected in carnivores in mainland Spain.

#### 3.2.2. Molecular Characterization of *Encephalitozoon cuniculi*

**Figure 3:**
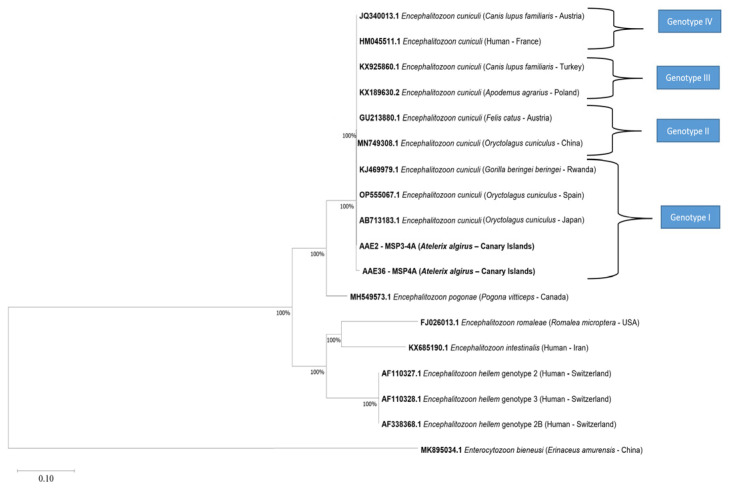
Phylogenetic relationships between partial sequences of the 16S rRNA gene, the complete internal transcribed spacer region (ITS), and the partial sequence of the 5.8S rRNA gene of *Encephalitozoon cuniculi* obtained in this study and known genotype sequences deposited in GenBank. The tree was constructed using the neighbor-joining method based on the genetic distance calculated using the Kimura 2-parameter model. Representative sequences for each *E. cuniculi* genotype (I–IV) were used. Accession numbers are shown in bold, and information concerning the host species and origin are shown in parentheses. *Enterocytozoon bieneusi* (MK895034.1) was used as an outgroup. There were a total of 211 positions in the final dataset.

Two of the thirty-six (5.6%) fecal samples were positive for *E. cuniculi*. Both sequences showed >99% homology with *E. cuniculi* sequences deposited in GenBank (AB713183.1, L13332.1, and OP555067.1). Phylogenetic analysis confirmed the identity of the isolates as *E. cuniculi* (bootstrap 100%) (Figure 3) and were identified as genotype I based on ITS sequence analysis.

#### 3.2.3. Geographical Distribution

Of the 16 genotyped *E. bieneusi* isolates, the most frequently detected was genotype AAE1 (81.25%; 13/16). It showed a wide distribution, being detected in seven municipalities in Tenerife and in the sampled municipality in Gran Canaria. In contrast, the genotype AAE2 (18.75%; 3/16) was detected only in two northern municipalities of Tenerife Island, El Sauzal, and Tacoronte.

*Encephalitozoon cuniculi*-positive hedgehogs were found in the northeastern zone of Tenerife, specifically in Santa Cruz de Tenerife and El Rosario.

Undetermined species were detected in Adeje and San Cristóbal de La Laguna; no other species were detected at these locations (Figure 1, Table 2).

## 4. Discussion

The present study constitutes the first report of microsporidia in the North African hedgehog (*A. algirus*), highlighting the presence of human-pathogenic microsporidia, *E. bieneusi* and *E. cuniculi*.

In Spain, *E. bieneusi* has been reported as the most frequent etiological agent of intestinal microsporidiosis in patients with human immunodeficiency virus (HIV) [21,22,23,24,25,26,27,28,29], as well as in non-HIV patients [30,31,32,33,34,35,36], and in sporadic cases of extraintestinal microsporidiosis [21,22,28,30,34,35,37]. However, cases of *Encephalitozoon* spp. have rarely been identified in Spain [29,32,38,39,40,41,42].

Data on microsporidia infection in patients from the Canary Islands are scarce, with only two reports of *E. bieneusi* in immunocompetent patients from Tenerife [35] and transplant recipients from Gran Canaria (genotype D) [36].

Regarding the zoonotic role of microsporidia, several animal hosts have been described as reservoirs of this group of parasites. To date, *E. bieneusi* is the most common species in Spain and has been detected in pet dogs [43,44,45,46], wild animals (lagomorphs, rodents, carnivores, and ungulates) [43,47,48,49,50,51,52], animals in urban environments (pigeons and cats) [46,53,54], farm animals (goats, rabbits, pigs, ostriches, cattle, and deer) [43,44,45,51,52,55,56] and animals in zoos (chimpanzees) [57]. In studies wherein genotyping was performed, most of the detected genotypes clustered within Group 1, suggesting zoonotic potential. *Encephalitozoon intestinalis* is the second most frequent species in animals. It has been reported in domestic cats [45], wildlife rabbits and hares [47,58], pigeons from parks [53], and farmed pigs and ostriches [45], whereas there are only a few reports of *E. hellem* [53,59] and *E. cuniculi* [50,60,61].

The prevalence obtained using the microscopic method in this study (100%; 36/36) differed from that obtained using nested-PCR (61.1%; 22/36), as reported in other studies [46,50]. This difference can be explained by the spontaneous extrusion of spores or low parasitic load, as suggested in the studies conducted by Izquierdo et al. [50] and Haro et al. [53], respectively.

The overall prevalence obtained in animals using the molecular methods in studies conducted in Spain with similar sample sizes ranged from 43.8% (14/32) in domestic dogs in Madrid [45], to 55.6% (15/27) in farmed pigs in Extremadura and Castile and León, [45] and 65.4% (17/26) in Iberian lynx in Andalusia [50], with *E. bieneusi* being most commonly detected in fecal samples in the latter studies. However, *E. bieneusi* was detected in 7 of the 14 PCR-positive dog samples and in 7 of the 15 PCR-positive pig samples compared to the 17 of the 22 nested-PCR-positive samples detected in this study.

In the case of the Iberian lynx, the results were in agreement with the positive samples obtained in this study for *E. bieneusi* (76.5%; 13/17 in lynxes vs. 77.3%; 17/22 in hedgehogs) and *E. cuniculi* (11.8%; 2/17 in lynxes vs. 9.1%; 2/22 in hedgehogs).

Considering the host species, the 47.2% (17/36) prevalence of *E. bieneusi* reported in *A. algirus* fecal samples is higher than that reported in a study conducted in China, which reported a prevalence of 9.8% (4/41) in *E. amurensis* intestine samples [10], but lower than the 70.0% (266/380) reported in fecal samples from farmed and pet *A. albiventris*, also conducted in China. The highest genetic diversity was recorded in *A. albiventris* with one known genotype, SCR05 (88.3%; 235/266) and 10 novel genotypes, GDH01 (3.4%; 9/266), GDH02 (0.8%; 3/266), and GDH03– GDH10 (one sample each) [11], followed by *E. amurensis* with four novel genotypes, EA1, EA2, EA3, and EA4 (one sample each) [10]. The population of *A. algirus* on the Canary Islands showed low genetic diversity and two novel genotypes, AAE1 and AAE2.

To the best of our knowledge, this is the first study to detect *E. cuniculi* in hedgehogs. The prevalence obtained (5.6%; 2/36) was similar to that recently reported in fecal samples from European rabbits in Tenerife (4.0%; 2/50) [61]. Genotype I of *E. cuniculi* has been the only genotype detected in animal hosts in Spain to date [60,61], and the species is less frequent in this country, with a few cases in humans [40,41,42]. However, molecular detection in water sources, in addition to serological analysis of domestic and wild animals, demonstrates the presence of this parasite in the environment [50,61,62].

Considering that the diet of hedgehogs is mainly based on Coleoptera [63] and numerous species of microsporidia have been described as insect parasites [64], the undetermined species detected in fecal samples of hedgehogs are suspected to be microsporidia species infecting invertebrate hosts. Other studies have also detected undetermined species using molecular methods [45,61,62].

The remaining human-pathogenic *Encephalitozoon* species, *E. intestinalis* and *E. hellem* were not detected in this study. A low prevalence of *E. intestinalis* has been reported in animals in Spain [45,47,53,58] and *E. hellem* in mammals worldwide [65].

Considering the high prevalence of *E. bieneusi* genotypes with zoonotic potential, veterinary control measures should be implemented to detect this pathogen, given that hedgehogs have been kept as pets on the Canary Islands [66] and could pose a risk to children who are most susceptible to microsporidiosis [67]. Despite the limited number of *E. cuniculi* cases detected in this study, the zoonotic risk should not be underestimated because symptomatic cases have been documented in humans [9].

## 5. Conclusions

This study constitutes the first report of microsporidia in fecal samples of the North African hedgehog (*A. algirus*). The overall prevalence of nested-PCR-confirmed samples was 61.1% (22/36), with *E. bieneusi* being the most common species, followed by the undetermined species and *E. cuniculi*. Two novel genotypes of *E. bieneusi* were identified, named AAE1 and AAE2, both clustered within Group 1, and the *E. cuniculi* isolates were identified as genotype I.

The results obtained in this study provide new data on the epidemiology of microsporidia on the Canary Islands (Spain), suggesting that zoonotic genotypes of human-pathogenic microsporidia circulate in the fauna of the islands, posing a risk to public and veterinary health.

## Figures and Tables

**Table 1:** Sequence differences in the internal transcribed spacer region of the rRNA gene of the novel genotype AAE1 compared to the closest matched sequences.

Genotype (Host)	Nucleotide Position (5′→ 3′) ^1^							
	31	32	51	52	53	86	131	155
AAE1 (*Atelerix algirus*)	A	T	G	T	-	G	G	A
HND-I (*Rhinopithecus bieti*)	G	T	G	T	A	G	G	A
EA1 (*Erinaceus amurensis*)	G	C	G	T	A	G	G	A
EA2 (*Erinaceus amurensis*)	G	T	-	-	A	G	G	A
EA3 (*Erinaceus amurensis*)	G	T	G	T	A	A	G	A
EA4 (*Erinaceus amurensis*)	G	T	G	T	A	G	G	G
S9 (*Vulpes vulpes*)	G	T	G	T	A	G	A	A

^1^ Nucleotide positions in the internal transcribed spacer region (ITS) of *Enterocytozoon bieneusi* (~243-bp). Hyphen indicates a deletion in this position.

**Table 2:** Geographical distribution of microsporidia species and genotypes identified in *Atelerix algirus* on the Canary Islands.

Location	Sample Size (*n*)	Nested-PCR Confirmed Samples (*n*)	*E. bieneusi* Genotypes (*n*)	*E. cuniculi* Genotypes (*n*)	Undetermined Species
Adeje	1	1	-	-	1
Arafo	1	-	-	-	-
Arona	7	6	AAE1 (5) Undetermined genotype (1) *	-	-
El Rosario	3	2	AAE1 (1)	I (1)	-
El Sauzal	1	1	AAE2 (1)	-	-
Granadilla de Abona	3	2	AAE1 (2)	-	-
Güímar	1	-	-	-	-
Icod de Los Vinos	1	-	-	-	-
Las Palmas de Gran Canaria	3	3	AAE1 (3)	-	-
San Cristóbal de La Laguna	7	2	-	-	2
San Miguel de Abona	1	-	-	-	-
Santa Cruz de Tenerife	5	3	AAE1 (2)	I (1)	-
Tacoronte	2	2	AAE2 (2)	-	-
TOTAL	36	22	17	2	3

* The sample was not successfully genotyped.

## Data Availability

Not applicable.

## Supplementary Materials

The following supporting information can be downloaded at: https://www.mdpi.com/article/10.3390/ani13111756/s1, Table S1: Microsporidia detected in hedgehog (*Atelerix algirus*) fecal samples and location in the Canary Islands (Spain).
