# Supplementary material for: High Prevalence of Microsporidia in the North African Hedgehog (Atelerix algirus) in the Canary Islands, Spain
Source: Animals (Basel). 2023 May 25;13(11):1756. doi: 10.3390/ani13111756 (PMC10251962; doi:10.3390/ani13111756)
Supplement: Supplementary file 1 [file animals-13-01756-s001.zip › animals-2379904-supplementary.pdf]

Table S1. Microsporidia detected in hedgehog (*Atelerix algirus*) fecal samples and location in the Canary Islands (Spain).

Sample ID	Location	Origin[1]	Sex	Microsporidia
AA1	Arona	WRC	-	*E. bieneusi*[2]
AA2	Santa Cruz de Tenerife	WRC	-	*E. cuniculi* (I)
AA4	San Cristóbal de La Laguna	WRC	-	-
AA5	San Cristóbal de La Laguna	WRC	-	-
AA6	Adeje	WRC	-	Undetermined
AA7	San Cristóbal de La Laguna	WRC	-	-
AA8	San Cristóbal de La Laguna	WRC	-	Undetermined
AA9	El Rosario	WRC	Male	-
AA10	Arafo	WRC	Male	-
AA11	Arona	WRC	Female	*E. bieneusi* (AAE1)
AA12	Santa Cruz de Tenerife	WRC	Male	-
AA13	Güímar	WRC	Male	-
AA14	Santa Cruz de Tenerife	WRC	-	*E. bieneusi* (AAE1)
AA15	Icod de Los Vinos	WRC	Female	-
AA16	Arona	WRC	-	*E. bieneusi* (AAE1)
AA17	San Cristóbal de La Laguna	WRC	Male	Undetermined
AA19	Granadilla de Abona	WRC	Male	*E. bieneusi* (AAE1)
AA20	El Sauzal	WRC	Male	*E. bieneusi* (AAE2)
AA21	Santa Cruz de Tenerife	WRC	Female	*E. bieneusi* (AAE1)
AA22	San Cristóbal de La Laguna	WRC	Male	-
AA23	Arona	WRC	Male	-
AA24	Granadilla de Abona	WRC	Female	-
AA25	Tacoronte	WRC	Female	*E. bieneusi* (AAE2)
AA26	Arona	WRC	Male	*E. bieneusi* (AAE1)
AA27	Arona	WRC	Male	*E. bieneusi* (AAE1)
AA28	San Miguel de Abona	WRC	Female	-
AA29	Arona	WRC	Male	*E. bieneusi* (AAE1)
AA30	Tacoronte	WRC	Female	*E. bieneusi* (AAE2)
AA31	Granadilla de Abona	WRC	Female	*E. bieneusi* (AAE1)
AA32	Las Palmas de Gran Canaria	Found dead	Female	*E. bieneusi* (AAE1)
AA33	Las Palmas de Gran Canaria	Found dead	-	*E. bieneusi* (AAE1)
AA34	Las Palmas de Gran Canaria	Found dead	Female	*E. bieneusi* (AAE1)
AA35	El Rosario	WRC	Female	*E. bieneusi* (AAE1)
AA36	El Rosario	WRC	-	*E. cuniculi* (I)
AA37	San Cristóbal de La Laguna	WRC	-	-
AA39	Santa Cruz de Tenerife	WRC	-	-

[1] WRC = wildlife recovery center; [2] The sample was not succesfully genotyped.
